# Reversible cardiac dysfunction in severe COVID‐19 infection, mechanisms and case report

**DOI:** 10.1111/echo.14807

**Published:** 2020-08-27

**Authors:** Chieh‐Ju Chao, Patrick A. DeValeria, Ayan Sen, Hong Lee, Dawn M Pedrotty, Bhavesh Patel, Reza Arsanjani, Tasneem Z. Naqvi

**Affiliations:** ^1^ Department of Cardiovascular Diseases Mayo Clinic Scottsdale AZ USA; ^2^ Department of Cardiovascular Surgery Mayo Clinic Scottsdale AZ USA; ^3^ Department of Critical Care Medicine Mayo Clinic Scottsdale AZ USA

**Keywords:** COVID‐19, myocarditis, speckle‐tracking strain, stress‐induced cardiomyopathy

## Abstract

A previously healthy 49‐year‐old male patient presented with COVID‐19 infection and required mechanical ventilation and extracorporeal membrane oxygenation due to severe hypoxemia. Echocardiography showed cardiac dysfunction with an apical sparing strain pattern, which rapidly normalized within a week. Apical sparing myocardial strain in patients with COVID‐19 infection may suggest reverse‐type stress cardiomyopathy.

## 1 INTRODUCTION

The novel COVID‐19 mainly attacks the respiratory system; however, the involvement of other end organs is common in critically ill patients. The most common symptoms of COVID‐19 infection are fever and cough, and 15% of patients may eventually require intensive care.^1^ Myocardial injury or infarction, myocarditis, congestive heart failure, arrhythmias, and cardiogenic shock have been reported with COVID‐19 infection.^2^, ^3^, ^4^ Among all patients with COVID‐19, myocardial injury was reported in more than 7% and 23% in critically ill patients.^5^ Although the exact mechanism of cardiac involvement in COVID‐19 remains unclear, the possible pathogenesis is believed to be related to direct cardiac involvement through the ACE2 signaling pathway.^6^, ^7^ Increased secretion of ACE2 in patients with underlying cardiovascular diseases may also explain the significantly increased risk of death in this group of patients.^8^, ^9^

Microvascular dysfunction, demand ischemia, plaque rupture, or cytokine storm triggered by an imbalanced response to type 1 and type 2 T helper cells are other potential mechanisms of cardiac injury. Given that ACE2 is also found on vascular endothelial cells, infection‐medicated vasculitis is also possible.^10^ Cytokine and inflammatory mediators can result in arterial inflammation, which could result in coronary plaque rupture. However, no reports yet associate SARS‐CoV‐2 with acute coronary syndrome (ACS). A case of presumably acute myocarditis due to COVID‐19 infection described LV apical hypokinesis,^11^ and the other two cases reported decreased LVEF.^12^, ^13^ Apical‐type stress‐induced cardiomyopathy was recently reported as a possible presentation of COVID‐19–related cardiac involvement.^14^ Herein, we discuss another potential presentation of cardiac injury in COVID 19 infection that occurred in a previously healthy patient who became critically ill with COVID‐19 infection.

## 2 HISTORY OF PRESENTATION

**Figure 1:**
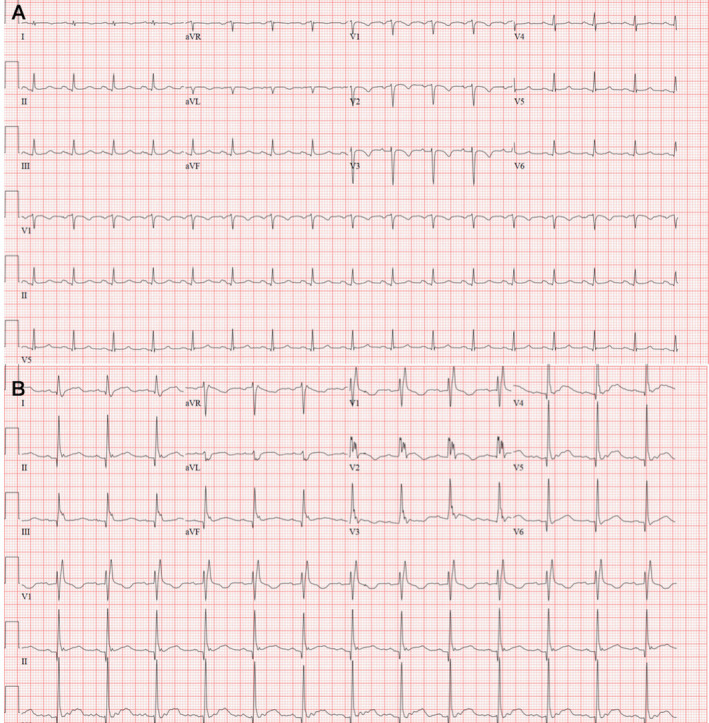
Baseline and follow‐up ECGs. Panel A. The initial ECG showed sinus tachycardia, narrow QRS, QTc interval of 467 ms, and T‐wave inversion in the precordial leads V1‐V3, I and aVL. Panel B. Three days later, a repeat ECG showed right bundle branch block, QTc of 539 ms, and mild diffuse ST elevation

**Figure 2:**
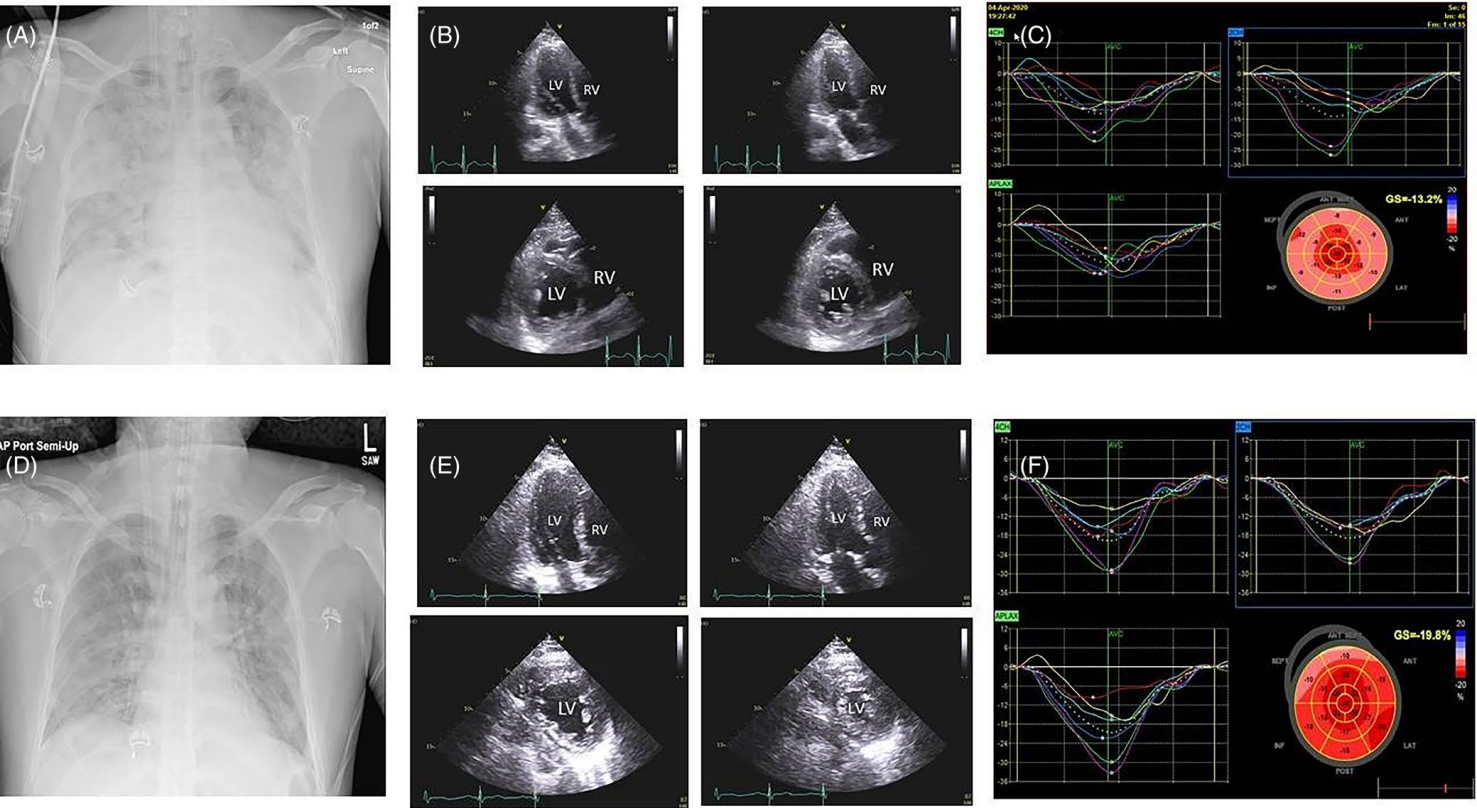
Panel A. Baseline chest X‐ray AP view showing diffuse lung parenchymal opacities consistent with acute respiratory distress syndrome. Panel B. A baseline 2D transthoracic echocardiographic (TTE) apical 3‐chamber view in end‐diastole (upper left image), end‐systole (upper right image), and parasternal short‐axis view at midventricular level in end‐diastole (lower left image) and end‐systole (lower right image). Increased left ventricular (LV) end‐systolic cavity size from basal to mid segments is shown (Video S1). Panel C. 3‐, 2‐, and 4‐chamber strain maps and bull's eye plot showing regional and global averaged LV longitudinal peak systolic strain of ‐ 13.2% with an apical sparing pattern. Panel D. Follow‐up chest X‐ray AP view showing marked improvement in ARDS. E. Follow‐up 2D TTE shows an apical 3‐chamber view in end‐diastole (upper left image), end‐systole (upper right image), and parasternal short‐axis view at midventricular level in end‐diastole (lower left image) and end‐systole (lower right image). The change of LV chamber size over the cardiac cycle suggests normalization of wall motion and LV ejection fraction (Video S2). Panel F. Follow‐up regional strain maps and global averaged LV longitudinal peak systolic strain of −19.8% with near‐normal strain pattern

A previously healthy 49‐year‐old male patient presented to a local emergency department with fever, cough, and pneumonia, and tested positive for COVID‐19. He was transferred to the intensive care unit and placed on lung‐protective ventilation with a diagnosis of acute respiratory distress syndrome (ARDS) based on chest X‐ray and severe hypoxemia. Transthoracic echocardiogram (TTE) showed a left ventricular ejection fraction (LVEF) of 50%. He then developed refractory hypoxemia. Our emergency transport team performed an urgent offsite veno‐venous (V‐V) extracorporeal membrane oxygenation (ECMO) placement and transferred the patient to our intensive care unit. Initial ventilator settings were FiO2 100%, TV 450 mL, PEEP 18 cmH2O, and V‐V ECMO settings were 2850 rpm and 3.5 L/min flow. Initial ECG showed narrow QRS, precordial T‐wave inversion, QTc of 467 ms (Figure 1A), and 3 days later showed right bundle branch block, prolonged QTc of 539 ms, and mild diffuse ST elevation (Figure 1B). The initial chest X‐ray revealed severe diffuse bilateral pulmonary infiltrates consistent with ARDS (Figure 2A). Central venous O2 saturation was 79%. Laboratory data showed initial: troponin 526 ng/L, creatinine kinase 506 U/L, NT‐proBNP 4,573 pg/mL, ferritin 3601 mcg/L, C‐reactive protein 104.9 mg/L (<8 mg/L), IL‐6 > 400 pg/mL (<1.8 pg/mL), creatinine 0.83 mg/dL, total bilirubin 1.5 mg/dL, AST 124 U/L, ALT 55 U/L, procalcitonin 6.28 ng/mL, and lactate 3 mmol/L. The initial TTE at our hospital showed mild to moderately reduced LVEF of 40% with marked hypokinesis of basal and mid segments and preserved wall motion of apical segments (Figure 2B and Video S1). Global averaged LV global longitudinal peak systolic strain (GLS) was −13.2% (normal ≥ negative than −18%) with preserved apical strain (Figure 2C).

Mixed venous saturation was not consistent with a diagnosis of cardiogenic shock. Vasopressin and norepinephrine were infused to maintain adequate mean arterial pressure for refractory hypotension from vasodilatory shock. Broad‐spectrum antibiotics were started for possible pneumonia. The patient received two doses of Tocilizumab for signs and biomarkers suggestive of cytokine release syndrome. Hydroxychloroquine and azithromycin were administered initially, however, discontinued after 2 days due to QTc prolongation (Figure 1B).

The patient was continued on V‐V ECMO, mechanical ventilation, and antibiotics, and his oxygen requirements progressively decreased to FiO2 40%, TV 200‐300 mL, and PEEP 10 cmH2O. Troponin had decreased to 41 ng/L, ferritin to 788 mcg/L, C‐reactive protein to 6.5 mg/L (<8 mg/L), and IL‐6 to 181 pg/mL (normal < 1.8 pg/mL).

Six days later, infiltrates started resolving on the chest X‐ray (Figure 2D), and a follow‐up TTE showed normalization of LVEF to 55% and marked improvement in regional wall motion abnormalities (Figure 2E and Video S2). LV GLS normalized to −19.8% (Figure 2F). The patient's pulmonary status continued to improve, tracheostomy was performed on day 10, and the patient was decannulated from V‐V ECMO on day 12. He remained culture negative.

## 3 DISCUSSION

In this case, LV wall motion abnormalities in a noncoronary distribution followed by rapid normalization of wall motion do not suggest ACS and are most consistent with reverse type of stress cardiomyopathy involving basal and mid‐LV segments as opposed to apical LV segments in the classical type of stress or Takotsubo cardiomyopathy. A recent report also described a typical takotsubo presentation in a COVID‐19–infected patient who required V‐A ECMO support^14^; a recent case series further reported a prevalence of stress cardiomyopathy of 4.2% in male COVID‐19 patients.^15^ Perhaps, the most compelling mechanism of myocardial injury in our patient is secondary to cytokine storm and a hyperinflammatory state that occurred in the later stage of the disease after direct viral cytotoxicity of the pulmonary system (ARDS) and multi‐organ dysfunction. This case demonstrated a unique echocardiographic strain pattern associated with rapidly reversible COVID‐19 myocardial dysfunction in a critically ill COVID‐19–positive patient. Published case reports have described various mechanisms of COVID‐19–related myocardial injury and are listed in Table 1. Apical stress cardiomyopathy has been reported but reverse stress cardiomyopathy has not been reported thus far.

**Table 1:** Echocardiographic features of COVID‐19–related myocardial injury in available case reports

	LV dimensions	LV wall motion	LVEF	Pericardial effusion	Diagnosis
Case 1^11^	Normal	Diffuse hypokinesis	40%	Circumferential, 11 mm (max)	Acute myopericarditis
Case 2^12^	58 mm	‐‐	27%	Trace, 2 mm (max)	Coronavirus fulminant myocarditis
Case 3^13^	61 mm	Diffuse dyskinesia	32%	No	Fulminant myocarditis
Case 4^14^	NA	Regional wall motion abnormality with apical ballooning	20%	No	Apical‐type stress‐induced cardiomyopathy

Myocardial inflammation and myocarditis can be detected by endomyocardial biopsy or by magnetic resonance imaging, which may show a variable pattern of Gadolinium enhancement.^16^ Our patient did not undergo magnetic resonance imaging or endomyocardial biopsy, as these tests were not feasible in this critically ill patient. The markedly elevated troponin upon presentation, development of marked conduction abnormality, and diffuse appearing mild ST elevation on follow‐up ECG may be suggestive of myopericarditis with apical sparing possibly from reduced apical distribution of myocardial ACE 2 receptors. However, rapid cardiac recovery without steroids is highly unusual in myocardial inflammation or infection. The apical sparing LV strain distribution pattern in our patient is commonly seen in cardiac amyloidosis and nonischemic cardiomyopathy,^17^ and has not been described in viral myocarditis.^18^ While apical hypokinesis or ballooning is the most common echo finding in stress cardiomyopathy,^14^ basal or mid segmental variant of stress cardiomyopathy ^19^ is not infrequent and is associated with basal to mid segmental hypokinesis with preserved apical wall motion and apical strain,^20^ mild ST elevation, QT prolongation, T‐wave inversion, and rapid recovery of LV function—all present in our patient. It is interesting to note that recovery of LV systolic function, although associated with improvement in pulmonary status, occurred prior to the complete resolution of ARDS. Cytokine storm and sympathetic surge that occurred early in the course of our patient's illness were the likely triggers of stress cardiomyopathy and marked troponin elevation.^10^ Improvement in cardiac function in our patient followed the administration of Tocilizumab, which is an IL‐6 inhibitor, improves cytokine release syndrome, and has also been reported to be effective in ARDS in a prior study.^21^ The effectiveness of Tocilizumab in treating COVID‐19–related acute myocarditis is unclear.^21^ Cases from China reported complete recovery of fulminant myocarditis treated with glucocorticoid and human immunoglobulin.^12^, ^13^ We did not need to use either of those therapies. Since Azithromycin and Hydroxychloroquine were given for a short period, and no steroids were administered, these likely did not contribute to the cardio‐pulmonary recovery.

## 4 CONCLUSION

We describe a patient with COVID‐19 infection who developed severe ARDS requiring mechanical ventilation and V‐V ECMO, and acute myocardial dysfunction with rapid recovery of cardiac function. The cardiac dysfunction was associated with apical sparing of wall motion and strain. It was rapidly reversible, suggesting basal to mid variant of stress cardiomyopathy as the probable etiology of LV dysfunction. Our findings suggest that inflammatory and cardiac biomarkers, as well as ECG and echocardiographic assessment, should be performed in patients who develop a severe illness due to COVID 19 infection. Speckle‐tracking strain imaging can provide insight into the mechanism of cardiac dysfunction, particularly in the absence of cardiac magnetic resonance imaging (CMR) or endomyocardial biopsy. Follow‐up echo assessment, along with strain imaging, may determine a change in cardiac function as well as its potential mechanism. Wall motion abnormalities in a noncoronary distribution either in the distal LV segments or in the basal LV segments along with rapid normalization of cardiac function and wall motion in the absence of steroid administration should raise the suspicion for an apical or basal variant of Takotsubo cardiomyopathy. Assessment of myocardial strain in patients with COVID‐19 infection can aid in the detection of preserved basal or apical wall motion. It may provide a clue to the presence of stress cardiomyopathy and its potential reversibility. Both apical type and reverse type of stress‐induced cardiomyopathy can develop in the setting of COVID‐19 infection and should be considered in the differential diagnosis of mechanism of myocardial injury in COVID‐19 infection. The use of myocardial strain is invaluable in this setting.

## DISCLOSURES

There are no relevant disclosures or conflicts of interest for this work by any of the authors.

## Supporting information

Video S1

Video S2
